# Supplementary material for: Chalcogen Vacancies Rule Charge Recombination in Pnictogen Chalcohalide Solar-Cell Absorbers
Source: ACS Energy Lett. 2025 Jun 30;10(7):3562–9. doi: 10.1021/acsenergylett.5c01267 (PMC12261329; doi:10.1021/acsenergylett.5c01267)
Supplement: Supplementary file 1 [file nz5c01267_si_001.pdf]

Supporting Information for "Chalcogen Vacancies Rule Charge Recombination in Pnictogen Chalcohalide Solar-Cell Absorbers"

Cibrán López,[1,2] Seán R. Kavanagh,[3] Pol Benítez,[1,2] Edgardo Saucedo,[2,4] Aron Walsh,[5,6] David O. Scanlon,[7] and Claudio Cazorla[1,2]

[1]*Departament de Física, Universitat Politècnica de Catalunya, 08034 Barcelona, Spain*
[2]*Barcelona Research Center in Multiscale Science and Engineering, Universitat Politècnica de Catalunya, 08019 Barcelona, Spain*
[3]*Harvard University Center for the Environment, Cambridge, Massachusetts 02138, United States*
[4]*Department of Electronic Engineering, Universitat Politècnica de Catalunya, 08034 Barcelona, Spain*
[5]*Thomas Young Centre and Department of Materials, Imperial College London, Exhibition Road, London SW7 2AZ, UK*
[6]*Department of Physics, Ewha Womans University, 52 Ewhayeodae-gil, Seodaemun-gu, Seoul 03760, South Korea*
[7]*School of Chemistry, University of Birmingham, Birmingham B15 2TT, UK*

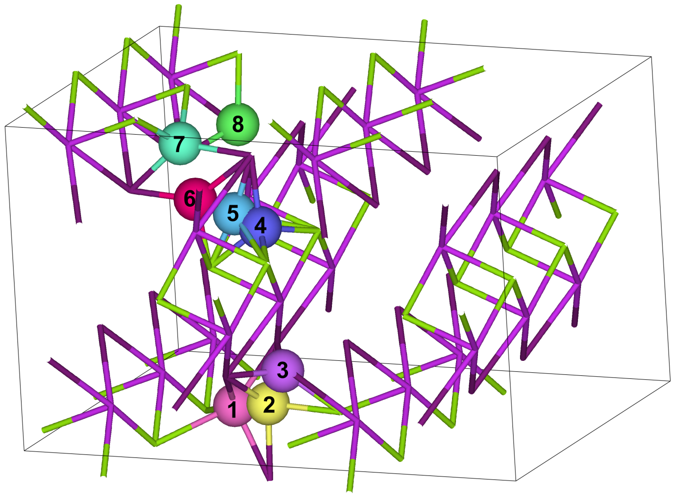

Supplementary Fig. 1. For interstitial defects, we initially evaluate their neutral states by sampling all inequivalent lattice sites for Bi, Se and I. Interstitials 1-8 for each species are represented in carnation, banana, lavender, orchid, sky, strawberry, spindrift and flora colors, respectively, according to the nomenclature used in the main text. Bi, Se and I atoms are represented with purple, green and grey sticks, respectively.

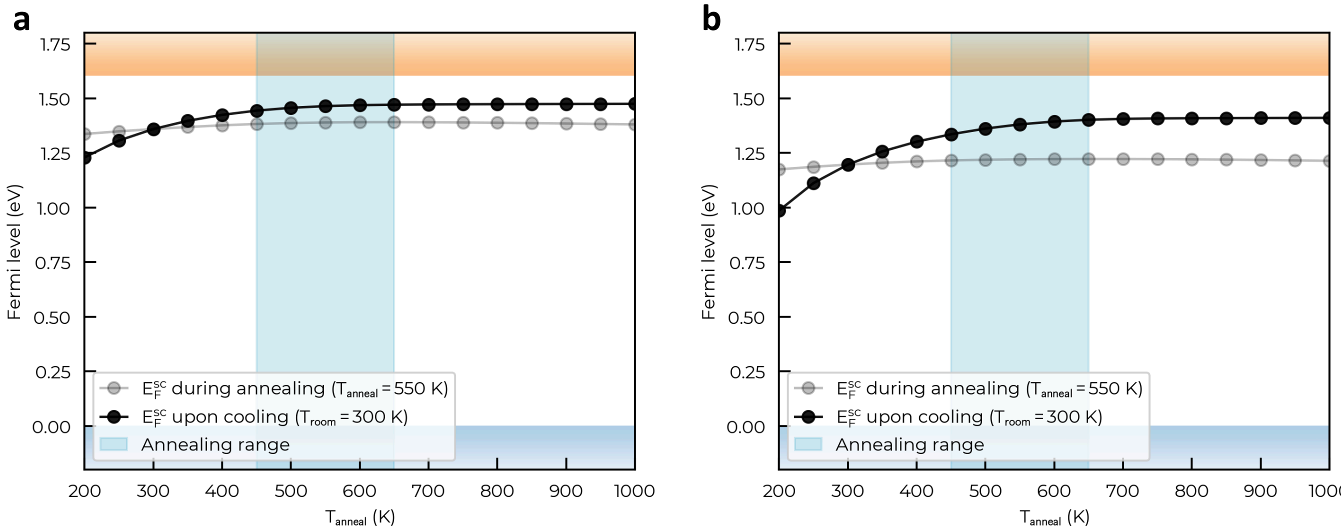

Supplementary Fig. 2. Temperature dependence of the Fermi level during annealing synthesis conditions for **a.** Se-poor and **b.** Bi-poor conditions.

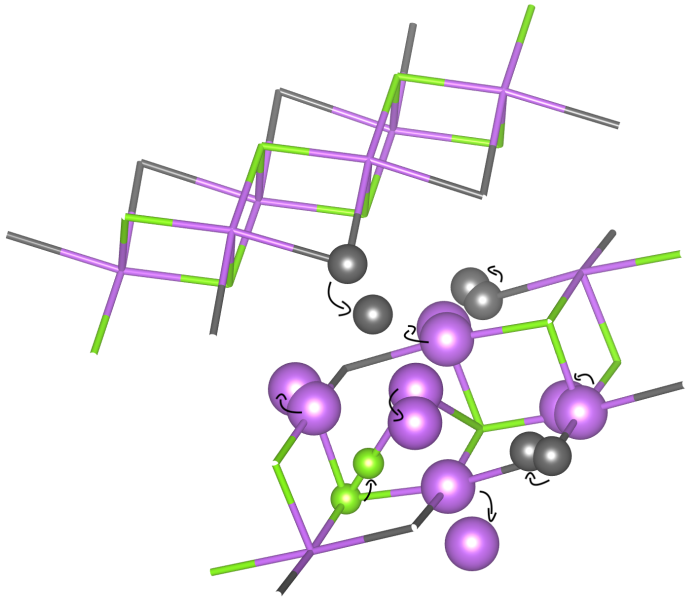

Supplementary Fig. 3. Bi_{Se} antisite undergoes a strong geometrical reconstruction from charge states -1 to +5. Only those atoms which differ at least 0.5 Å between both charge states are represented. Arrows show how atoms migrate from the -1 to the +5 charge states. Bi, Se and I atoms are represented with purple, green and grey spheres, respectively

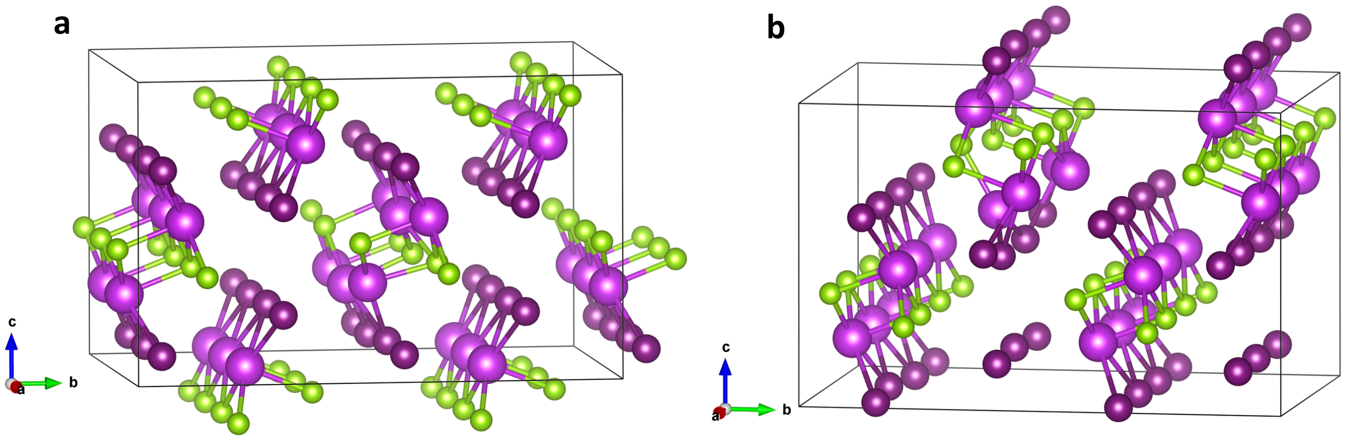

Supplementary Fig. 4. **a.** Vacancies (e.g., V_{Se}^{0}) and **b.** interstitials (e.g., $Bi_{i,4}^{+1}$) require significant structural adjustments within the affected column, exhibit mostly high formation energies.

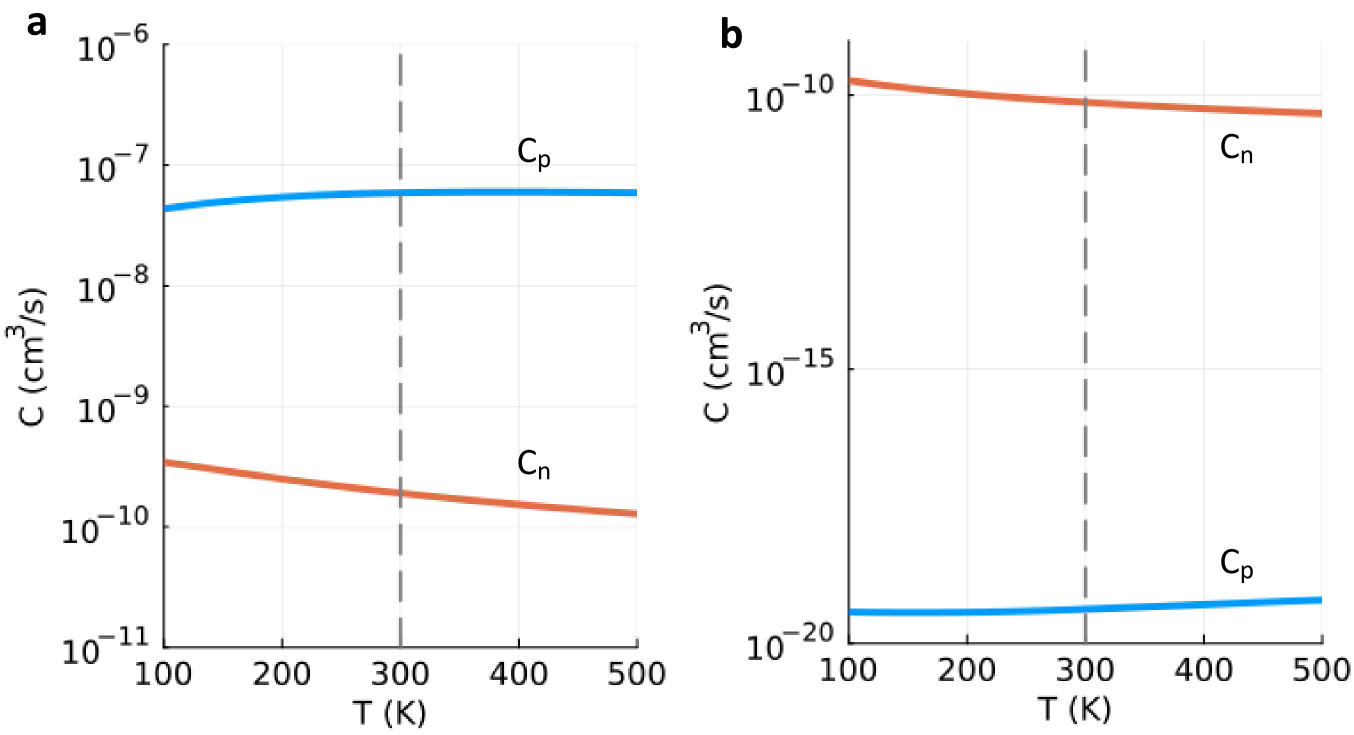

Supplementary Fig. 5. Electron (n)/hole (p) non-radiative capture coefficients C_x for **a.** V_{Se} $(0/+1)$ and **b.** Bi_{Se} $(0/+1)$ expressed as a function of temperature.

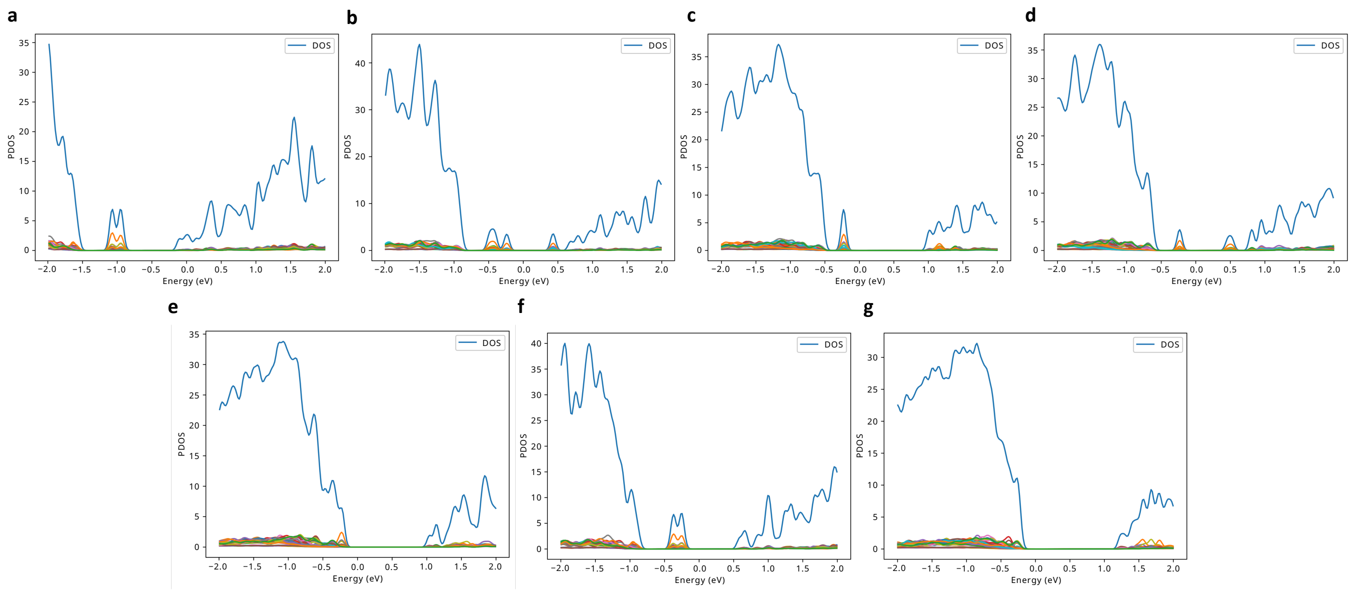

Supplementary Fig. 6. Partial contributions to the density of electronic states for the Bi_{Se} defect with a charge state of a) -2, b) -1, c) 0, d) +1, e) +2, f) +3 and g) +4 (in units of e). Bi, Se and I electronic contributions are represented with blue, green and orange lines, respectively.

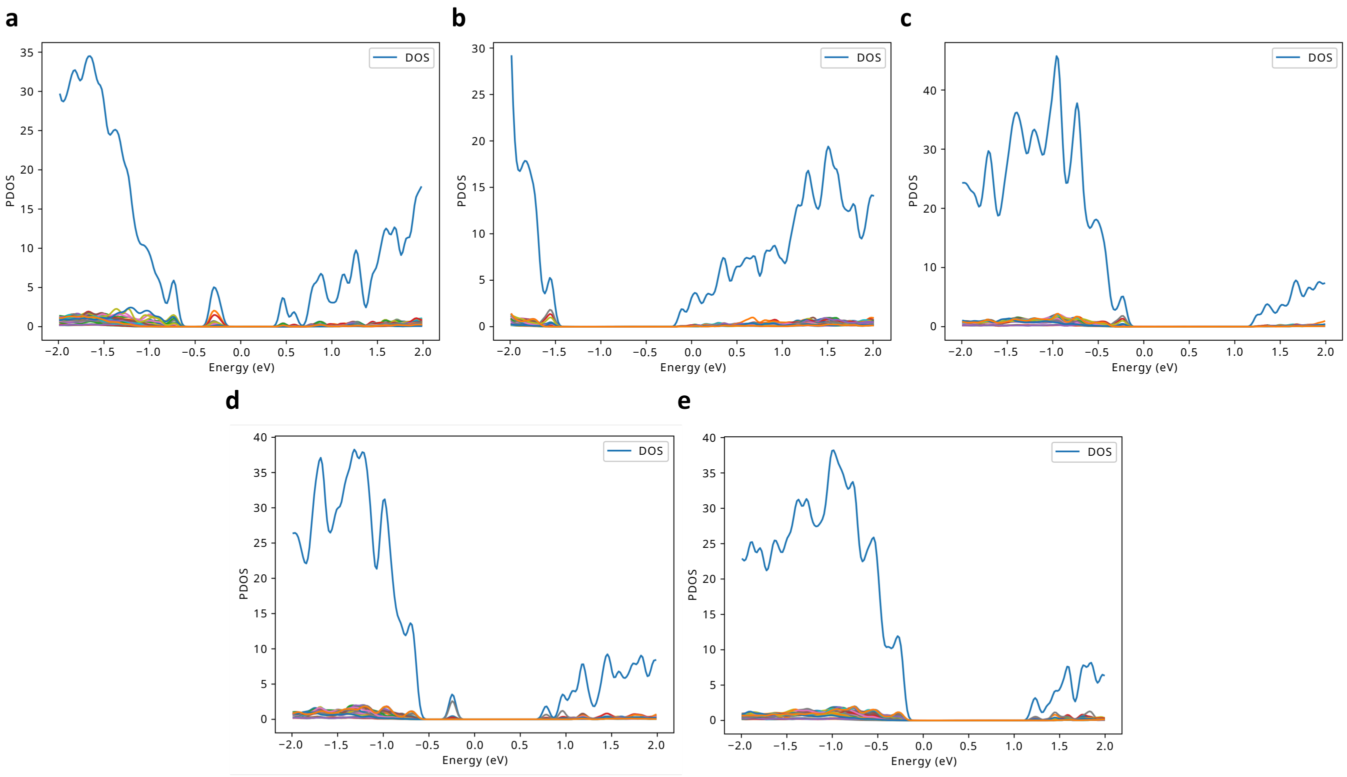

Supplementary Fig. 7. Partial contributions to the density of electronic states for the V_{Se} defect with a charge state of a) -2, b) -1, c) 0, d) +1 and e) +2 (in units of e). Bi, Se and I electronic contributions are represented with blue, green and orange lines, respectively.

	Bi	Se	I
BiSeI-Se-BiI_3	-0.97	0.00	-0.42
BiSeI-Bi_2Se_3-Se	-0.89	0.31	-0.51
BiSeI-Bi-BiI_3	0.00	-0.65	-0.75
BiSeI-Bi_2Se_3-Bi	0.00	-0.59	0.80

Supplementary Table I. Chemical potential (in units of eV) limits in BiSeI, with BiSeI, Bi, Se, I, Bi_2Se_3 and BiI_3 crystallizing into structures with the $Pnma$, $R\overline{3}m$, $P3$, $Cmcm$, $R\overline{3}m$ and $R\overline{3}$ space groups, respectively.

Defect	q	$E_T[X^q] - E_T[bulk]$	qE_{VBM}	$\sum n_i E_{i,ref}$	$\sum n_i \mu_i^{Bi-poor}$	$\sum n_i \mu_i^{Se-poor}$	$E_{corr}[X^q]$	$E^f[X^q]^{Bi-poor}$	$E^f[X^q]^{Se-poor}$
V_{Bi}^{+3}	+3	-1.37	7.23	-5.72	-0.97	0.00	-0.42	-1.24	-0.27
V_{Bi}^{+2}	+2	1.92	4.82	-5.72	-0.97	0.00	-0.28	-0.23	0.74
V_{Bi}^{+1}	+1	4.53	2.41	-5.72	-0.97	0.00	-0.06	0.19	1.16
V_{Bi}^{0}	0	8.36	0.00	-5.72	-0.97	0.00	0.00	1.67	2.64
V_{Bi}^{-1}	-1	11.17	-2.41	-5.72	-0.97	0.00	0.21	2.28	3.25
V_{Bi}^{-2}	-2	15.19	-4.82	-5.72	-0.97	0.00	0.57	4.25	5.22
V_{Bi}^{-3}	-3	18.45	-7.23	-5.72	-0.97	0.00	1.05	5.58	6.55
V_{Se}^{+2}	+2	-0.73	4.82	-4.63	0.00	-0.65	-0.16	-0.70	-1.35
V_{Se}^{+1}	+1	2.95	2.41	-4.63	0.00	-0.65	-0.00	-0.70	0.08
V_{Se}^{0}	0	6.10	0.00	-4.63	0.00	-0.65	0.00	1.47	0.82
V_{Se}^{-1}	-1	10.09	-2.41	-4.63	0.00	-0.65	0.02	3.07	2.42
V_{Se}^{-2}	-2	14.11	-4.82	-4.63	0.00	-0.65	0.21	4.87	4.22
V_{I}^{+1}	+1	0.32	2.41	-2.26	-0.42	-0.75	-0.04	0.01	-0.32
V_{I}^{0}	0	4.13	0.00	-2.26	-0.42	-0.75	0.00	1.46	1.13
V_{I}^{-1}	-1	7.98	-2.41	-2.26	-0.42	-0.75	0.22	3.11	2.78

Supplementary Table II. Calculated formation energies at $E_F = 0$ for Bi/Se-poor synthesis conditions ($E^f[X^q]^{Bi/Se-poor}$) of all native point defects in BiSeI under Bi-poor conditions, showing all contributing terms in the formation energy equation: defect-bulk supercell raw DFT energy difference ($E_{Defect} - E_{Bulk}$), VBM Fermi level reference (qE_{VBM}), elemental reference energy contributions ($\sum n_i E_{i,ref}$), formal chemical potential contributions for Bi/Se-poor synthesis conditions ($\sum n_i \mu_i^{Bi/Se-poor}$) and finite-size charge corrections (E_{corr}). All energies are given in units of eV.

Defect	q	$E_T[X^q] - E_T[bulk]$	qE_{VBM}	$\sum n_i E_{i,ref}$	$\sum n_i \mu_i^{Bi-poor}$	$\sum n_i \mu_i^{Se-poor}$	$E_{corr}[X^q]$	$E^f[X^q]^{Bi-poor}$	$E^f[X^q]^{Se-poor}$
Bi_{Se}^{+5}	+5	-17.44	12.06	1.09	0.97	-0.65	0.16	-3.16	-4.79
Bi_{Se}^{+4}	+4	-13.82	9.65	1.09	0.97	-0.65	0.23	-1.89	-3.51
Bi_{Se}^{+3}	+3	-10.59	7.23	1.09	0.97	-0.65	0.57	-0.72	-2.34
Bi_{Se}^{+2}	+2	-6.53	4.82	1.09	0.97	-0.65	0.38	0.73	-0.90
Bi_{Se}^{+1}	+1	-3.34	2.41	1.09	0.97	-0.65	0.15	1.28	-0.34
Bi_{Se}^{0}	0	0.36	0.00	1.09	0.97	-0.65	0.00	2.42	0.80
Bi_{Se}^{-1}	-1	3.83	-2.41	1.09	0.97	-0.65	-0.06	3.42	1.80
Bi_{Se}^{-2}	-2	7.89	-4.82	1.09	0.97	-0.65	-0.19	4.94	3.31
Bi_{I}^{+5}	+5	-18.32	12.06	3.46	0.55	-0.75	-0.59	-2.84	-4.14
Bi_{I}^{+4}	+4	-15.65	9.64	3.46	0.55	-0.75	-0.06	-2.05	-3.35
Bi_{I}^{+3}	+3	-11.96	7.23	3.46	0.55	-0.75	0.26	-0.46	-1.75
Bi_{I}^{+2}	+2	-8.61	4.82	3.46	0.55	-0.75	0.27	0.50	-0.80
Bi_{I}^{+1}	+1	-4.99	2.41	3.46	0.55	-0.75	0.09	1.53	0.23
Bi_{I}^{0}	0	-1.57	0.00	3.46	0.55	-0.75	0.00	2.44	1.15
Bi_{I}^{-1}	-1	2.26	-2.41	3.46	0.55	-0.75	0.08	3.95	2.65
Bi_{I}^{-2}	-2	6.13	-4.82	3.46	0.55	-0.75	0.17	5.49	4.19
Se_{Bi}^{+5}	+5	-10.97	12.06	-1.09	-0.97	0.65	-0.88	-1.86	-0.23
Se_{Bi}^{+4}	+4	-8.66	9.65	-1.09	-0.97	0.65	-0.16	-1.24	0.39
Se_{Bi}^{+3}	+3	-5.29	7.23	-1.09	-0.97	0.65	-0.52	-0.64	0.99
Se_{Bi}^{+2}	+2	-2.92	4.82	-1.09	-0.97	0.65	-0.14	-0.30	1.33
Se_{Bi}^{+1}	+1	-0.45	2.41	-1.09	-0.97	0.65	-0.03	-0.12	1.50
Se_{Bi}^{0}	0	3.01	0.00	-1.09	-0.97	0.65	0.00	0.95	2.57
Se_{Bi}^{-1}	-1	6.35	-2.41	-1.09	-0.97	0.65	0.13	2.00	3.63
Se_{Bi}^{-2}	-2	10.32	-4.82	-1.09	-0.97	0.65	0.05	3.48	5.10
Se_{I}^{+5}	+5	-15.72	12.06	2.38	-0.42	-0.10	-0.51	-2.22	-1.90
Se_{I}^{+4}	+4	-13.19	9.65	2.38	-0.42	-0.10	-0.40	-1.99	-1.67
Se_{I}^{+3}	+3	-10.66	7.23	2.38	-0.42	-0.10	0.21	-1.27	-0.94
Se_{I}^{+2}	+2	-6.02	4.82	2.38	-0.42	-0.10	-0.13	0.62	0.95
Se_{I}^{+1}	+1	-4.38	2.41	2.38	-0.42	-0.10	0.03	0.02	0.34
Se_{I}^{0}	0	-0.75	0.00	2.38	-0.42	-0.10	0.00	1.20	1.53
Se_{I}^{-1}	-1	. 2.08	-2.41	2.38	-0.42	-0.10	0.14	1.76	2.09
Se_{I}^{-2}	-2	6.04	-4.82	2.38	-0.42	-0.10	0.04	3.22	3.54
I_{Bi}^{+5}	+5	-9.01	12.06	-3.46	-0.55	0.75	-0.36	-1.33	-0.03
I_{Bi}^{+4}	+4	-6.45	9.64	-3.46	-0.55	0.75	0.29	-0.53	0.77
I_{Bi}^{+3}	+3	-3.17	7.23	-3.46	-0.55	0.75	0.16	0.21	1.51
I_{Bi}^{+2}	+2	-0.59	4.82	-3.46	-0.55	0.75	0.05	0.27	1.57
I_{Bi}^{+1}	+1	2.60	2.41	-3.46	-0.55	0.75	-0.00	0.99	2.29
I_{Bi}^{0}	0	5.65	0.00	-3.46	-0.55	0.75	0.00	1.63	2.93
I_{Bi}^{-1}	-1	9.33	-2.41	-3.46	-0.55	0.75	0.06	2.97	4.27
I_{Bi}^{-2}	-2	12.68	-4.82	-3.46	-0.55	0.75	0.15	4.00	5.30
I_{Se}^{+5}	+5	-12.78	12.06	-2.38	0.42	0.10	-0.45	-3.13	-3.45
I_{Se}^{+4}	+4	-9.76	9.65	-2.38	0.42	0.10	-0.63	-2.70	-3.02
I_{Se}^{+3}	+3	-7.18	7.23	-2.38	0.42	0.10	0.15	-1.74	-2.07
I_{Se}^{+2}	+2	-3.80	4.82	-2.38	0.42	0.10	0.02	-0.91	-1.23
I_{Se}^{+1}	+1	-1.26	2.41	-2.38	0.42	0.10	0.09	-0.71	-1.03
I_{Se}^{0}	0	2.75	0.00	-2.38	0.42	0.10	0.00	0.80	0.47
I_{Se}^{-1}	-1	. 7.19	-2.41	-2.38	0.42	0.10	-0.05	2.78	2.45
I_{Se}^{-2}	-2	11.09	-4.82	-2.38	0.42	0.10	-0.23	4.09	3.77

Supplementary Table III. Calculated formation energies at $E_F = 0$ for Bi/Se-poor synthesis conditions ($E^f[X^q]^{Bi/Se-poor}$) of all native point defects in BiSeI under Bi-poor conditions, showing all contributing terms in the formation energy equation: defect-bulk supercell raw DFT energy difference ($E_{Defect} - E_{Bulk}$), VBM Fermi level reference (qE_{VBM}), elemental reference energy contributions ($\sum n_i E_{i,ref}$), formal chemical potential contributions for Bi/Se-poor synthesis conditions ($\sum n_i \mu_i^{Bi/Se-poor}$) and finite-size charge corrections (E_{corr}). All energies are given in units of eV.

Defect	q	$E_T[X^q] - E_T[bulk]$	qE_{VBM}	$\sum n_i E_{i,ref}$	$\sum n_i \mu_i^{Bi-poor}$	$\sum n_i \mu_i^{Se-poor}$	$E_{corr}[X^q]$	$E^f[X^q]^{Bi-poor}$	$E^f[X^q]^{Se-poor}$
$Bi_{i,1}^{+3}$	+3	-16.11	7.23	5.72	0.97	0.00	0.94	-1.25	-2.22
$Bi_{i,1}^{+2}$	+2	-12.31	4.82	5.72	0.97	0.00	0.55	-0.25	-1.22
$Bi_{i,1}^{+1}$	+1	-8.97	2.41	5.72	0.97	0.00	0.20	0.34	-0.63
$Bi_{i,1}^{0}$	0	-4.74	0.00	5.72	0.97	0.00	0.00	1.95	0.98
$Bi_{i,2}^{+3}$	+3	-16.03	7.23	5.72	0.97	0.00	0.57	-1.54	-2.51
$Bi_{i,2}^{+2}$	+2	-12.31	4.82	5.72	0.97	0.00	0.54	-0.25	-1.22
$Bi_{i,2}^{+1}$	+1	-8.97	2.41	5.72	0.97	0.00	0.20	0.34	-0.63
$Bi_{i,2}^{0}$	0	-4.23	0.00	5.72	0.97	0.00	0.00	2.46	1.49
$Bi_{i,3}^{+3}$	+3	-16.03	7.23	5.72	0.97	0.00	0.58	-1.53	-2.50
$Bi_{i,3}^{+2}$	+2	-12.30	4.82	5.72	0.97	0.00	0.54	-0.25	-1.22
$Bi_{i,3}^{+1}$	+1	-8.60	2.41	5.72	0.97	0.00	0.19	0.69	-0.28
$Bi_{i,3}^{+4}$	0	-4.23	0.00	5.72	0.97	0.00	0.00	2.46	1.49
$Bi_{i,4}^{+3}$	+3	-15.78	7.23	5.72	0.97	0.00	0.89	-0.96	-1.94
$Bi_{i,4}^{+2}$	+2	-12.24	4.82	5.72	0.97	0.00	0.46	-0.27	-1.24
$Bi_{i,4}^{+1}$	+1	-8.96	2.41	5.72	0.97	0.00	0.21	0.35	-0.62
$Bi_{i,4}^{0}$	0	-4.74	0.00	5.72	0.97	0.00	0.00	1.95	0.98
$Bi_{i,5}^{0}$	0	-4.16	0.00	5.72	0.97	0.00	0.00	2.53	1.56
$Bi_{i,6}^{0}$	0	-4.16	0.00	5.72	0.97	0.00	0.00	2.53	1.56
$Bi_{i,7}^{0}$	0	-4.16	0.00	5.72	0.97	0.00	0.00	2.53	1.56
$Bi_{i,8}^{0}$	0	-4.16	0.00	5.72	0.97	0.00	0.00	2.53	1.56

Supplementary Table IV. Calculated formation energies at $E_F = 0$ for Bi/Se-poor synthesis conditions ($E^f[X^q]^{Bi/Se-poor}$) of all native point defects in BiSeI under Bi-poor conditions, showing all contributing terms in the formation energy equation: defect-bulk supercell raw DFT energy difference ($E_{Defect} - E_{Bulk}$), VBM Fermi level reference (qE_{VBM}), elemental reference energy contributions ($\sum n_i E_{i,ref}$), formal chemical potential contributions for Bi/Se-poor synthesis conditions ($\sum n_i \mu_i^{Bi/Se-poor}$) and finite-size charge corrections (E_{corr}). All energies are given in units of eV.

Defect	q	$E_T[X^q] - E_T[bulk]$	qE_{VBM}	$\sum n_i E_{i,ref}$	$\sum n_i \mu_i^{Bi-poor}$	$\sum n_i \mu_i^{Se-poor}$	$E_{corr}[X^q]$	$E^f[X^q]^{Bi-poor}$	$E^f[X^q]^{Se-poor}$
$Se_{i,9}^{+6}$	+6	-21.56	14.47	4.63	0.00	0.65	-0.05	-2.54	-1.89
$Se_{i,9}^{+5}$	+5	-19.29	12.06	4.63	0.00	0.65	0.39	-2.21	-1.56
$Se_{i,9}^{+4}$	+4	-15.94	9.65	4.63	0.00	0.65	0.26	-1.40	-0.75
$Se_{i,9}^{+3}$	+3	-12.91	7.23	4.63	0.00	0.65	0.50	-0.55	0.10
$Se_{i,9}^{+2}$	+2	-10.35	4.82	4.63	0.00	0.65	0.39	-0.51	0.14
$Se_{i,9}^{+1}$	+1	-7.04	2.41	4.63	0.00	0.65	0.14	0.14	0.79
$Se_{i,9}^{0}$	0	-3.70	0.00	4.63	0.00	0.65	0.00	0.93	1.58
$Se_{i,9}^{-1}$	-1	0.35	-2.41	4.63	0.00	0.65	-0.09	2.48	3.13
$Se_{i,9}^{-2}$	-2	4.86	-4.82	4.63	0.00	0.65	0.04	4.70	5.35
$Se_{i,10}^{+6}$	+6	-20.54	14.47	4.63	0.00	0.65	-0.56	-2.00	-1.35
$Se_{i,10}^{+5}$	+5	-19.08	12.06	4.63	0.00	0.65	0.21	-2.18	-1.53
$Se_{i,10}^{+4}$	+4	-16.46	9.65	4.63	0.00	0.65	0.05	-2.13	-1.48
$Se_{i,10}^{+3}$	+3	-13.30	7.23	4.63	0.00	0.65	0.21	-1.23	-0.58
$Se_{i,10}^{+2}$	+2	-10.32	4.82	4.63	0.00	0.65	0.35	-0.52	0.13
$Se_{i,10}^{+1}$	+1	-6.69	2.41	4.63	0.00	0.65	0.17	0.52	1.17
$Se_{i,10}^{0}$	0	-3.87	0.00	4.63	0.00	0.65	0.00	0.76	1.41
$Se_{i,10}^{-1}$	-1	0.44	-2.41	4.63	0.00	0.65	-0.13	2.53	3.18
$Se_{i,10}^{-2}$	-2	4.86	-4.82	4.63	0.00	0.65	0.03	4.70	5.35
$Se_{i,11}^{+6}$	+6	-21.04	14.47	4.63	0.00	0.65	0.27	-1.67	-1.02
$Se_{i,11}^{+5}$	+5	-18.49	12.06	4.63	0.00	0.65	0.09	-1.71	-1.06
$Se_{i,11}^{+4}$	+4	-15.86	9.65	4.63	0.00	0.65	-0.61	-2.20	-1.55
$Se_{i,11}^{+3}$	+3	-12.93	7.23	4.63	0.00	0.65	0.23	-0.83	-0.18
$Se_{i,11}^{+2}$	+2	-10.32	4.82	4.63	0.00	0.65	0.35	-0.52	0.13
$Se_{i,11}^{+1}$	+1	-6.69	2.41	4.63	0.00	0.65	0.17	0.52	1.17
$Se_{i,11}^{0}$	0	-3.76	0.00	4.63	0.00	0.65	0.00	0.87	1.52
$Se_{i,11}^{-1}$	-1	0.35	-2.41	4.63	0.00	0.65	-0.09	2.48	3.13
$Se_{i,11}^{-2}$	-2	4.70	-4.82	4.63	0.00	0.65	-0.11	4.40	5.05
$Se_{i,12}^{+6}$	+6	-23.42	14.47	4.63	0.00	0.65	-0.41	-4.73	-4.08
$Se_{i,12}^{+5}$	+5	-20.18	12.06	4.63	0.00	0.65	0.03	-3.46	-2.81
$Se_{i,12}^{+4}$	+4	-16.91	9.65	4.63	0.00	0.65	0.29	-2.35	-1.70
$Se_{i,12}^{+3}$	+3	-13.90	7.23	4.63	0.00	0.65	0.46	-1.58	-0.93
$Se_{i,12}^{+2}$	+2	-10.88	4.82	4.63	0.00	0.65	0.28	-1.14	-0.49
$Se_{i,12}^{+1}$	+1	-7.12	2.41	4.63	0.00	0.65	0.15	0.08	0.73
$Se_{i,12}^{0}$	0	-3.76	0.00	4.63	0.00	0.65	0.00	0.87	1.52
$Se_{i,12}^{-1}$	-1	0.28	-2.41	4.63	0.00	0.65	-0.14	2.36	3.01
$Se_{i,12}^{-2}$	-2	4.64	-4.82	4.63	0.00	0.65	0.02	4.46	5.11

Supplementary Table V. Calculated formation energies at $E_F = 0$ for Bi/Se-poor synthesis conditions ($E^f[X^q]^{Bi/Se-poor}$) of all native point defects in BiSeI under Bi-poor conditions, showing all contributing terms in the formation energy equation: defect-bulk supercell raw DFT energy difference ($E_{Defect} - E_{Bulk}$), VBM Fermi level reference (qE_{VBM}), elemental reference energy contributions ($\sum n_i E_{i,ref}$), formal chemical potential contributions for Bi/Se-poor synthesis conditions ($\sum n_i \mu_i^{Bi/Se-poor}$) and finite-size charge corrections (E_{corr}). All energies are given in units of eV.

Defect	q	$E_T[X^q]-E_T[bulk]$	qE_{VBM}	$\sum n_i E_{i,ref}$	$\sum n_i \mu_i^{Bi-poor}$	$\sum n_i \mu_i^{Se-poor}$	$E_{corr}[X^q]$	$E^f[X^q]^{Bi-poor}$	$E^f[X^q]^{Se-poor}$
$Se_{i,13}^{+6}$	+6	-22.70	14.47	4.63	0.00	0.65	-0.60	-4.21	-3.56
$Se_{i,13}^{+5}$	+5	-19.65	12.06	4.63	0.00	0.65	0.26	-2.70	-2.05
$Se_{i,13}^{+4}$	+4	-16.03	9.65	4.63	0.00	0.65	-0.09	-1.84	-1.19
$Se_{i,13}^{+3}$	+3	-13.90	7.23	4.63	0.00	0.65	0.46	-1.57	-0.93
$Se_{i,13}^{+2}$	+2	-10.36	4.82	4.63	0.00	0.65	0.33	-0.58	0.07
$Se_{i,13}^{+1}$	+1	-6.74	2.41	4.63	0.00	0.65	0.11	0.42	1.07
$Se_{i,13}^{0}$	0	-3.88	0.00	4.63	0.00	0.65	0.00	0.76	1.41
$Se_{i,13}^{-1}$	-1	0.28	-2.41	4.63	0.00	0.65	-0.14	2.36	3.01
$Se_{i,13}^{-2}$	-2	4.64	-4.82	4.63	0.00	0.65	-0.12	4.33	4.98
$Se_{i,14}^{+6}$	+6	-22.01	14.47	4.63	0.00	0.65	0.33	-2.58	-1.93
$Se_{i,14}^{+5}$	+5	-18.85	12.06	4.63	0.00	0.65	0.35	-1.81	-1.16
$Se_{i,14}^{+4}$	+4	-16.11	9.65	4.63	0.00	0.65	0.89	-0.94	-0.30
$Se_{i,14}^{+3}$	+3	-13.16	7.23	4.63	0.00	0.65	0.35	-0.94	-0.29
$Se_{i,14}^{+2}$	+2	-10.11	4.82	4.63	0.00	0.65	0.33	-0.33	0.32
$Se_{i,14}^{+1}$	+1	-7.04	2.41	4.63	0.00	0.65	0.13	0.13	0.78
$Se_{i,14}^{0}$	0	-3.71	0.00	4.63	0.00	0.65	0.00	0.93	1.58
$Se_{i,14}^{-1}$	-1	0.35	-2.41	4.63	0.00	0.65	-0.09	2.48	3.13
$Se_{i,14}^{-2}$	-2	4.71	-4.82	4.63	0.00	0.65	-0.09	4.43	5.08
$Se_{i,15}^{+6}$	+6	-21.59	14.47	4.63	0.00	0.65	-0.63	-3.12	-2.47
$Se_{i,15}^{+5}$	+5	-18.90	12.06	4.63	0.00	0.65	-0.10	-2.31	-1.66
$Se_{i,15}^{+4}$	+4	-16.70	9.65	4.63	0.00	0.65	0.59	-1.83	-1.18
$Se_{i,15}^{+3}$	+3	-13.14	7.23	4.63	0.00	0.65	0.41	-0.87	-0.22
$Se_{i,15}^{+2}$	+2	-10.37	4.82	4.63	0.00	0.65	0.34	-0.57	0.08
$Se_{i,15}^{+1}$	+1	-7.04	2.41	4.63	0.00	0.65	0.13	0.14	0.78
$Se_{i,15}^{0}$	0	-3.88	0.00	4.63	0.00	0.65	0.00	0.75	1.40
$Se_{i,15}^{-1}$	-1	0.35	-2.41	4.63	0.00	0.65	-0.09	2.48	3.13
$Se_{i,15}^{-2}$	-2	4.70	-4.82	4.63	0.00	0.65	-0.11	4.40	5.05
$Se_{i,16}^{+6}$	+6	-21.31	14.47	4.63	0.00	0.65	-0.72	-2.92	-2.28
$Se_{i,16}^{+5}$	+5	-18.99	12.06	4.63	0.00	0.65	-0.92	-3.22	-2.57
$Se_{i,16}^{+4}$	+4	-16.10	9.65	4.63	0.00	0.65	0.39	-1.43	-0.78
$Se_{i,16}^{+3}$	+3	-13.00	7.23	4.63	0.00	0.65	0.45	-0.68	-0.03
$Se_{i,16}^{+2}$	+2	-10.56	4.82	4.63	0.00	0.65	0.30	-0.80	-0.16
$Se_{i,16}^{+1}$	+1	-7.13	2.41	4.63	0.00	0.65	0.15	0.06	0.71
$Se_{i,16}^{0}$	0	-3.88	0.00	4.63	0.00	0.65	0.00	0.75	1.40
$Se_{i,16}^{-1}$	-1	0.44	-2.41	4.63	0.00	0.65	-0.13	2.53	3.18
$Se_{i,16}^{-2}$	-2	4.86	-4.82	4.63	0.00	0.65	0.03	4.70	5.35

Supplementary Table VI. Calculated formation energies at $E_F = 0$ for Bi/Se-poor synthesis conditions ($E^f[X^q]^{Bi/Se-poor}$) of all native point defects in BiSeI under Bi-poor conditions, showing all contributing terms in the formation energy equation: defect-bulk supercell raw DFT energy difference ($E_{Defect}-E_{Bulk}$), VBM Fermi level reference (qE_{VBM}), elemental reference energy contributions ($\sum n_i E_{i,ref}$), formal chemical potential contributions for Bi/Se-poor synthesis conditions ($\sum n_i \mu_i^{Bi/Se-poor}$) and finite-size charge corrections (E_{corr}). All energies are given in units of eV.

Defect	q	$E_T[X^q]-E_T[bulk]$	qE_{VBM}	$\sum n_i E_{i,ref}$	$\sum n_i \mu_i^{Bi-poor}$	$\sum n_i \mu_i^{Se-poor}$	$E_{corr}[X^q]$	$E^f[X^q]^{Bi-poor}$	$E^f[X^q]^{Se-poor}$
$I^0_{i,17}$	0	-0.90	0	2.26	0.42	0.75	0	1.78	2.11
$I^0_{i,18}$	0	-0.92	0	2.26	0.42	0.75	0	1.76	2.08
$I^0_{i,19}$	0	-0.92	0	2.26	0.42	0.75	0	1.76	2.08
$I^0_{i,20}$	0	-0.92	0	2.26	0.42	0.75	0	1.76	2.08
$I^0_{i,21}$	0	-0.93	0	2.26	0.42	0.75	0	1.75	2.07
$I^0_{i,22}$	0	-0.91	0	2.26	0.42	0.75	0	1.77	2.10
$I^0_{i,23}$	0	-0.93	0	2.26	0.42	0.75	0	1.75	2.07
$I^0_{i,24}$	0	-0.90	0	2.26	0.42	0.75	0	1.78	2.10

Supplementary Table VII. Calculated formation energies at $E_F = 0$ for Bi/Se-poor synthesis conditions ($E^f[X^q]^{Bi/Se-poor}$) of all native point defects in BiSeI under Bi-poor conditions, showing all contributing terms in the formation energy equation: defect-bulk supercell raw DFT energy difference ($E_{Defect}-E_{Bulk}$), VBM Fermi level reference (qE_{VBM}), elemental reference energy contributions ($\sum n_i E_{i,ref}$), formal chemical potential contributions for Bi/Se-poor synthesis conditions ($\sum n_i \mu_i^{Bi/Se-poor}$) and finite-size charge corrections (E_{corr}). All energies are given in units of eV.

Defect	Charge transition	ΔQ (amu$^{1/2}$Å)	ΔE (eV)	ΔE_p (eV)	ΔE_n (eV)	C_p (cm^3/s)	C_n (cm^3/s)	σ_p (cm^2)	σ_n (cm^2)
V_{Se}	-2/-1	33.64	0.61	0.09	0.81	0	$8.90 \cdot 10^{-21}$	0	$9.97 \cdot 10^{-28}$
V_{Se}	-1/0	1.95	0.00	11.90	0.00	$2.80 \cdot 10^{-20}$	$6.71 \cdot 10^{-9}$	$1.51 \cdot 10^{-27}$	$7.52 \cdot 10^{-16}$
V_{Se}	0/+1	15.77	0.87	2.36	0.01	$5.89 \cdot 10^{-8}$	$1.91 \cdot 10^{-10}$	$3.18 \cdot 10^{-15}$	$2.14 \cdot 10^{-17}$
V_{Se}	+1/+2	16.44	0.26	1.12	0.08	$2.98 \cdot 10^{-20}$	$1.24 \cdot 10^{-9}$	$1.61 \cdot 10^{-27}$	$1.39 \cdot 10^{-16}$
Bi_{Se}	-2/-1	1.58	0.13	7.93	0.13	$1.15 \cdot 10^{-23}$	$5.53 \cdot 10^{-10}$	$6.18 \cdot 10^{-31}$	$6.19 \cdot 10^{-17}$
Bi_{Se}	-1/0	8.45	0.70	3.30	0.10	$8.83 \cdot 10^{-21}$	$5.88 \cdot 10^{-10}$	$4.76 \cdot 10^{-28}$	$6.58 \cdot 10^{-17}$
Bi_{Se}	0/+1	12.00	0.46	1.93	0.00	$4.21 \cdot 10^{-20}$	$7.47 \cdot 10^{-11}$	$2.27 \cdot 10^{-27}$	$8.36 \cdot 10^{-18}$
Bi_{Se}	+1/+2	16.20	3.83	3.15	2.78	$1.34 \cdot 10^{-16}$	$3.09 \cdot 10^{-23}$	$7.25 \cdot 10^{-24}$	$3.47 \cdot 10^{-30}$
Bi_{Se}	+2/+3	45.05	69.10	68.50	68.90	$5.64 \cdot 10^{-38}$	$4.63 \cdot 10^{-38}$	$3.04 \cdot 10^{-45}$	$5.18 \cdot 10^{-45}$
Bi_{Se}	+3/+4	21.58	14.41	0.00	1.17	0	$1.38 \cdot 10^{-11}$	0	$7.42 \cdot 10^{-19}$
Bi_{Se}	+4/+5	33.69	1.00	0.18	0.67	$1.48 \cdot 10^{-43}$	$4.12 \cdot 10^{-19}$	$8.00 \cdot 10^{-51}$	$4.61 \cdot 10^{-26}$

Supplementary Table VIII. Calculated capture coefficients for each charge-carrier process involving V_{Se} and Bi_{Se}, being ΔE the transition energy above the VBM, and key parameters used to calculate the carrier capture coefficients in each transition. $\Delta E_{n/p}$ are the energy barriers for electron and hole capture processes, respectively. $C_{n/p}$ and $\sigma_{n/p}$ are the capture coefficients and cross sections, respectively. The electron and hole thermal velocities are $8.93 \cdot 10^6$ cm/s and $1.86 \cdot 10^7$ cm/s, respectively.

	X	Y	Z
ϵ	58.43	16.20	27.02
ϵ_i	45.65	5.98	15.62
ϵ_∞	12.78	10.22	11.40

Supplementary Table IX. Dielectric constant (total ϵ, and lattice ϵ_i and optical ϵ_∞ responses) with respect to the conventional unit cell directions X, Y, Z in BiSeI.

	X	Y	Z
m_e	0.61	0.52	0.29
m_h	1.47	0.31	0.38

Supplementary Table X. Electron and hole effective masses with respect to the conventional unit cell directions X, Y, Z in BiSeI.

SUPPLEMENTARY DISCUSSION

For Bi_{Se}, a strongly localized defect-bound electron polaron emerges at the charge state −2. This polaron is characterized by a favorable ionization energy of −0.20 eV (i.e., the energy difference between the charge-transition level and CBM). Small ionization energies significantly enhance polaron formation, while high ionization energies contribute to prolonged polaron lifetimes [1]. The lattice distortion associated with this polaron is primarily localized around the Bi ion. However, at higher charge states +4 and +5 (+5 corresponds to the fully-ionised charge state, thus there is no excess charge to localise), the charge becomes delocalized, causing the polaron to vanish (even though the lattice distortion remains apparent).

The analysis of V_{Se} reveals the presence of two types of polarons: a bipolaron with a charge state of −2, and a polaron with a charge state of +1 (Supplementary Fig. 4). The bipolaron is notably shared between the two Bi atoms adjacent to the vacancy, with a favorable ionization energy of 0.08 eV, indicating its likely formation. Conversely, the polaron with a charge state of +1 is centered around the Bi atom nearest to the vacancy. However, its high ionization energy of 1.05 eV indicates that its formation is not probable.

To evaluate the potential impact of polarons on the PV performance of BiSeI, we indirectly assessed their spatial extent using the Fröhlich coupling constant, α^F. This parameter quantifies the strength of the interactions between a charge carrier and the field of longitudinal optical (LO) phonons in polarizable materials (Methods). We obtained values of $\alpha^F_h = 1.4$ for holes and $\alpha^F_e = 1.1$ for electrons. Interestingly, these values are significantly lower than those reported for other chalcogenides, such as Sb_2Se_3 ($\alpha^F_h = 2.1$, $\alpha^F_e = 1.3$) and Sb_2S_3 ($\alpha^F_h = 2.0$, $\alpha^F_e = 1.6$) [2], and Bi-based semiconductors like $NaBiS_2$ ($\alpha^F_h = 2.9$, $\alpha^F_e = 1.4$) and $AgBiS_2$ ($\alpha^F_h = 1.6$, $\alpha^F_e = 1.1$) [3, 4]. Such results can be explained by BiSeI having a small static dielectric constant (Supplementary Table 9) and elevated LO phonon frequency ($\omega_{LO} = 19.68$ THz). The electron and hole effective masses are listed in Supplementary Table 10.

Polarons can be found looking at charge localizations around specific atoms. We represent contribution of each atom to the partial density on electronic states for Bi_{Se} (Supplementary Fig. 6) and V_{Se} (Supplementary Fig. 7).

SUPPLEMENTARY METHODS

The static dielectric tensor (ε_{stat}) included both electronic (ε_∞) and ionic (ε_0) contributions. For the computation of the ionic (lattice response) contribution, the dynamical matrix was constructed from a finite-differences approach, employing the PBEsol functional. For the electronic (optical response) contribution, the HSEsol+SOC functional was used, with a $8 \times 4 \times 3$ Γ-centered **k**-point mesh (Supplementary Table 9).

Exploration of the potential energy surface. Conventional approaches to generating defect configurations [5] from pristine cells fail to find many lowering-energy conformations, which might have a crucial effect in the conclusions. Therefore, once a defect is generated (e.g., extracting a bismuth atom), we look for distortions of the initial lattice configuration to locally explore the potential energy surface. These distortions were generated with the `ShakeNBreak` software package [6, 7].

Initially, all the trial defect configurations were relaxed using Γ-point reciprocal space sampling and the HSEsol+D3 functional. Only the minimum-energy configurations were kept. Next, the ionic relaxations were repeated considering a larger **k**-point grid of $2 \times 1 \times 2$ (Γ-centered). After that, the relaxations were repeated considering SOC corrections. Finally, single-point energy calculations were performed using the previously-converged electronic wavefunctions and equilibrium structures. Non-spherical contributions to the gradient of the density within the PAW spheres were taken into account to improve numerical accuracy.

Point-defect formation energies. The formation energy of a point defect with charge q, D^q, can be expressed as [8]:

$$E_{\mathrm{f}}(D^q) = E_T(D^q) - E_T(\text{pristine}) + \\ + qE_F - \sum_i n_i \mu_i + E_{corr}(D^q) \ , \tag{1}$$

where $E_T(D^q)$ and E_T(pristine) are the static energies of defected and pristine supercells (energy per formula unit), respectively, μ_i corresponds to chemical potential of species i (this is, the energy required to extract one single atom), n_i the number of extracted atoms (positive or negative if extracted or added to the pristine cell, respectively), E_F the Fermi energy (energy needed to extract an electron), and E_{corr} the finite-size corrections based on spurious interactions between charged defects due to the periodic boundary conditions.

Here we considered two different contributions to the finite-size energy correction: point-charge (due to the spurious electrostatic interactions of a defect with its images) and band-alignment corrections (charged defects spuriously change the electrostatic potential of the system). Both corrections are computed together from an extension of the Freysoldt-Neugebauer-Van de Walle [8] correction scheme to anisotropic materials [9], as implemented in the `doped` defect simulation package [10] (Supplementary Tables 2–7).

Fröhlich coupling constant. The Fröhlich (or polaron) coupling constant for electron and holes reads:

$$\alpha^F_{(e,h)} = \frac{e^2}{4\pi\epsilon_0\hbar}\left(\frac{1}{\varepsilon_\infty} - \frac{1}{\varepsilon_{stat}}\right)\sqrt{\frac{m_{(e,h)}}{2\hbar\omega_{LO}}} \ , \tag{2}$$

where ϵ_0 is the vacuum permittivity, $\hbar$ is the reduced Planck's constant, $m_{(e,h)}$ is the effective mass of electron or hole, and ω_{LO} is the effective longitudinal optical phonon frequency taken as the average over all Γ-point modes weighted by the dipole moment they produce [11] (additional technical details can be found in the Supplementary Discussion).

Defect-limited efficiency. The nonradiative recombination activity has been estimated from the electron and hole capture coefficients for each charge state of the defect, using the `CarrierCapture.jl` software package [12, 13].

Within the employed formalism, the maximum defect-limited photovoltaic efficiency [14] under incident radiation spectrum Φ is:

$$\eta = \max_V \left(\frac{JV}{q\int_0^\infty E\Phi(E)dE}\right) \ , \tag{3}$$

where q is the electron charge and J the maximum defect-limited current density:

$$J(W,V) = J_{SC}(W) + J_0^{rad}(W,V) + J_0^{nonrad}(W,V) \ , \tag{4}$$

J_{SC}, J_0^{rad} are the short-circuit and saturation currents, respectively. While these two terms lead the annihilation of charges due to radiative recombination, J_0^{nonrad} takes into account the non-radiative recombination:

$$J_{SC}(W) = q\int_0^\infty a(E,W)\Phi(E)dE \tag{5}$$

$$\begin{aligned} J_0^{rad}(W,V) = q\frac{2\pi}{c^2h^3}\left(1 - e^{\frac{qV}{k_BT}}\right) \times \\ \times \int_0^\infty a(E,W)\left(e^{\frac{E}{k_BT}} - 1\right)^{-1} E^2 dE \end{aligned} \tag{6}$$

$$J_0^{nonrad}(W,V) = -qWR^{SRH}(V) \ , \tag{7}$$

where a is the absorptivity of the system (detailed balance limit assuming that each photon generates an electron-hole pair) and:

$$R_{SRH} \approx \sum \Delta n/p \ N_T C_{n/p} \ , \tag{8}$$

where Δn, Δp, and N_T denote excess concentrations of electrons, holes, and concentration of defects, respectively, with the summation over all independent recombination centers in the system. The carrier capture coefficient [12, 15] ($C_{n/p}$) can be expressed using the electron-phonon coupling (W_{ct}) and the overlap of phonon wave functions ($\langle\zeta_{cm}|\Delta Q|\zeta_{tn}\rangle$), which is given by:

$$\begin{aligned} C_{n/p} = \Omega\frac{2\pi}{\hbar}|W_{ct}|^2\sum_{m,n} w_m\langle\zeta_{cm}|\Delta Q|\zeta_{tn}\rangle^2 \times \\ \times\delta(\Delta E_{n/p} + \epsilon_{cm} - \epsilon_{tn}) \ , \end{aligned} \tag{9}$$

with Ω the volume of the supercell, g the degeneracy of the defect, ζ the phonon wave function, ΔQ an effective configuration coordinate for the phonon wave functions, and the subscripts c and t the free carrier and trap states, respectively. In this formalism, the temperature-dependence is determined by the thermal occupation number w_m of the initial vibrational state. The `TLC` software package [15] was used to estimate the effect of carrier-capture kinetics on photovoltaic efficiency.

SUPPLEMENTARY REFERENCES

[1] R. L. Z. Hoye, J. Hidalgo, R. A. Jagt, J.-P. Correa-Baena, T. Fix, and J. L. MacManus-Driscoll. The role of dimensionality on the optoelectronic properties of oxide and halide perovskites, and their halide derivatives. *Adv. Energy Mater.*, 12:2100499, 2022.

[2] X. Wang, A. M. Ganose, S. R. Kavanagh, and A. Walsh. Band versus polaron: Charge transport in antimony chalcogenides. *ACS Energy Letters*, 7:2954–2960, 2022.

[3] Y. Wang, S. R. Kavanagh, I. Burgués-Ceballos, A. Walsh, D. O. Scanlon, and G. Konstantatos. Cation disorder engineering yields agbis2 nanocrystals with enhanced optical absorption for efficient ultrathin solar cells. *Nature Photonics*, 16:235–241, 2022.

[4] Yi-T. Huang, S. R. Kavanagh, M. Righetto, M. Rusu, I. Levine, T. Unold, S. J. Zelewski, A. J. Sneyd, K. Zhang, L. Dai, A. J. Britton, J. Ye, J. Julin, M. Napari, Z. Zhang, J. Xiao, M. Laitinen, L. Torrente-Murciano, S. D. Stranks, A. Rao, L. M. Herz, David O. Scanlon, A. Walsh, and R. L. Z. Hoye. Strong absorption and ultrafast localisation in nabis2 nanocrystals with slow charge-carrier recombination. *Nature Communications*, 13:4960, 2022.

[5] C. Freysoldt, B. Grabowski, T. Hickel, J. Neugebauer, G. Kresse, A. Janotti, and C. G. Van de Walle. First-principles calculations for point defects in solids. *Rev. Mod. Phys.*, 86:253–305, 2014.

[6] I. Mosquera-Lois, S. R. Kavanagh, A. Walsh, and D. O. Scanlon. Shakenbreak: Navigating the defect configurational landscape. *JOSS*, 7:4817, 2022.

[7] I. Mosquera-Lois, S. R. Kavanagh, A. Walsh, and D. O. Scanlon. Identifying the ground state structures of point defects in solids. *npj Comput. Mater.*, 9:25, 2023.

[8] C. Freysoldt, J. Neugebauer, and C. G. Van de Walle. Fully ab initio finite-size corrections for charged-defect supercell calculations. *Phys. Rev. Lett.*, 102:016402, 2009.

[9] Y. Kumagai and F. Oba. Electrostatics-based finite-size corrections for first-principles point defect calculations. *Phys. Rev. B*, 89:195205, 2014.

[10] S. R. Kavanagh, A. G. Squires, A. Nicolson, I. Mosquera-Lois, A. M. Ganose, B. Zhu, K. Brlec, and D. O. Walsh, A. and. Scanlon. doped: Python toolkit for robust and repeatable charged defect supercell calculations. *Journal of Open Source Software*, 9:6433, 2024.

[11] A. M. Ganose, J. Park, A. Faghaninia, R. Woods-Robinson, K. A. Persson, and A. Jain. Efficient calculation of carrier scattering rates from first principles. *Nature Communications*, 12:2222, 2021.

[12] A. Alkauskas, Q. Yan, and C. G. Van de Walle. First-principles theory of nonradiative carrier capture via multiphonon emission. *Phys. Rev. B*, 90:075202, 2014.

[13] S. Kim, S. N. Hood, P. van Gerwen, L. D. Whalley, and A. Walsh. Carriercapture.jl: Anharmonic carrier capture. *JOSS*, 5:2102, 2020.

[14] N. Dahan, A. Jehl, J. F. Guillemoles, D. Lincot, N. Naghavi, and J.-J. Greffet. Using radiative transfer equation to model absorption by thin cu(in,ga)se2 solar cells with lambertian back reflector. *Opt. Express*, 21:2563–2580, 2013.

[15] S. Kim, J. A. Márquez, T. Unold, and A. Walsh. Upper limit to the photovoltaic efficiency of imperfect crystals from first principles. *Energy Environ. Sci.*, 13:1481–1491, 2020.
